# Characterizing novel endogenous retroviruses from genetic variation inferred from short sequence reads

**DOI:** 10.1038/srep15644

**Published:** 2015-10-23

**Authors:** Tobias Mourier, Sarah Mollerup, Lasse Vinner, Thomas Arn Hansen, Kristín Rós Kjartansdóttir, Tobias Guldberg Frøslev, Torsten Snogdal Boutrup, Lars Peter Nielsen, Eske Willerslev, Anders J. Hansen

**Affiliations:** 1 Centre for GeoGenetics, Museum of Natural History of Denmark, University of Copenhagen, Copenhagen, Denmark; 2 Section for Virology, National Veterinary Institute, Technical University of Denmark, Frederiksberg, Denmark; 3 Department for Autoimmunology and Biomarkers, Statens Serum Institut, Copenhagen, Denmark

## Abstract

From Illumina sequencing of DNA from brain and liver tissue from the lion, *Panthera leo*, and tumor samples from the pike-perch, *Sander lucioperca*, we obtained two assembled sequence contigs with similarity to known retroviruses. Phylogenetic analyses suggest that the pike-perch retrovirus belongs to the epsilonretroviruses, and the lion retrovirus to the gammaretroviruses. To determine if these novel retroviral sequences originate from an endogenous retrovirus or from a recently integrated exogenous retrovirus, we assessed the genetic diversity of the parental sequences from which the short Illumina reads are derived. First, we showed by simulations that we can robustly infer the level of genetic diversity from short sequence reads. Second, we find that the measures of nucleotide diversity inferred from our retroviral sequences significantly exceed the level observed from Human Immunodeficiency Virus infections, prompting us to conclude that the novel retroviruses are both of endogenous origin. Through further simulations, we rule out the possibility that the observed elevated levels of nucleotide diversity are the result of co-infection with two closely related exogenous retroviruses.

The advent of next-generation sequencing (NGS) technologies has facilitated virus discovery without prior knowledge of specific viral sequences and has resulted in the discovery of a multitude of novel viruses^1^,^2^,^3^,^4^. NGS techniques typically produce large amounts of short sequence reads that are subsequently assembled into larger contiguous sequences (contigs)^5^. These contigs can then be compared against a database of known viral sequences allowing for considerable sequence divergence. The single stranded RNA genome of retroviruses is reverse transcribed to double-stranded DNA and integrated into the host genome upon infection. Thus, in cases where retroviral sequence contigs are identified from non-model organisms with no available reference genome sequence, a challenge exists in determining whether the retrovirus is of endogenous or exogenous origin.

An exogenous retrovirus may become endogenous if it is inserted in the germline and subsequently inherited in a Mendelian manner. Endogenous retroviruses may continue to proliferate within the genome similarly to other transposable genetic elements^6^, generating multiple genomic copies. An endogenous retrovirus inserted into the genome of an embryo will be present in all tissues in the fully developed organism, and if fixed within a population, will be carried by all individuals regardless of health conditions. In pathogen discovery it is therefore essential to be able to distinguish endogenous retroviruses from exogenous retroviruses.

Although successfully proliferating endogenous retroviruses often have lost the envelope gene^7^, the presence of an envelope gene is not evidence for a retrovirus being exogenous^8^. Further, as assembled contigs from NGS studies will often only represent a subset of the complete viral genome, these may not contain the complete repertoire of retroviral genes. The dissimilar evolutionary histories of exogenous and endogenous retroviruses are expected to result in different levels of genetic variation in viral sequences from within an individual (Fig. 1). If the insertion of a retrovirus in germline cells is of relatively ancient origin, numerous nucleotide changes will accumulate between intra-genomic copies of the endogenous retrovirus. Certain mutations may render copies less efficient in transposing, and for some transposable elements only a subset of elements are responsible for the majority of new transposition events^9^,^10^. Importantly, if an insertion of an endogenous retroviral sequence is not deleterious to the host, this copy may – unless being entirely lost by random genetic drift–remain in the genome despite being rendered immobile by accumulating mutations. Over time, this will result in a highly diversified population of endogenous retroviral sequences within the genome. In contrast, the population of exogenous retroviruses within a chronically infected host will have derived from a single, very recent ancestor. Individual infecting viruses are under strong negative selection, and will, unless successfully colonizing new cells, be removed from the population of viral sequences. One would therefore expect to be able to distinguish an exogenous retrovirus infection from an endogenous retrovirus family of a certain age by the level of genetic variation between individual viral sequences (Fig. 1).

Here, we present two retroviral contig sequences from lion, *Panthera leo*, and pike-perch, *Sander lucioperca*, that are identified through the assembly of short sequence reads produced from NGS. The contigs were produced from a larger virus discovery experiment, which included the enrichment of nucleic acid protected by viral capsids as previously described^11^,^12^. Although this will ideally alter the ratio between viral sequences and host genome, samples still present a mix of foreign viral sequences and host DNA. Therefore, virion enriched samples may still contain integrated proviral DNA, and the finding of retroviral sequences poses the question as to whether these are derived from newly integrated exogenous retroviruses or from endogenous retroviruses in the host genome. Usually, answering this question involves additional time- and labour-intensive experimental work, as well as access to sample material from additional tissue and/or individuals. We therefore wished to develop an approach based exclusively on short sequence reads. Through simulations, we show that the genetic variation of parental retroviral sequences can be inferred from short sequence reads, and we compare the genetic variation of our retroviruses to the variation observed in infections with known exogenous retroviruses.

## Results and Discussion

### Novel retroviral sequences

Illumina sequencing of DNA from samples from the lion, *P. leo*, and the pike-perch, *S. lucioperca* resulted in two assembled contigs with similarity to known retroviruses. The two contigs, henceforth referred to simply as ‘lion’ and ‘perch’, were of lengths 5976 and 2903 base pairs (bp), respectively. Protein domains corresponding to Gag-protein, protease, reverse transcriptase, RNase H, and integrase were identified in open reading frames in the lion contig, whereas the perch contig only contained RNase H and integrase-encoding domains (Supplementary Fig. S1). A phylogenetic analysis of integrase sequences from a range of retroviruses formed—with the exception of gamma- and epsilon retroviruses – monophyletic clusters of retrovirus subfamilies (Fig. 2). In this phylogeny, the lion sequence falls within the gammaretroviruses and the perch sequence within the epsilonretroviruses (Fig. 2). The perch retrovirus sequence groups with two endogenous retroviruses, ZFERV and ZFERV-2, discovered in the zebra fish, *Danio rerio*, and an exogenous retrovirus, ASSBSV, causing swim bladder carcinoma in salmon^13^. The ZFERV retrovirus was not found in other closely related species and thus appeared to be restricted to *Danio rerio*^14^. Noticeably, the perch retrovirus sequence does not cluster with the epsilonretrovirus, WDSV associated with the development of tumors in the closely related American walleye^15^. At the amino acid level, the perch contig showed the highest level of identity to ZFERV-2 (59% and 51% identity for RNase H and integrase, respectively) and a lower level of identity to ZERV (39% and 40% in the two domains, respectively) (Supplementary Fig. S2). The lion contig is phylogenetically placed close to Feline Leukemia Virus (FeLV) for which endogenous variants are known^16^,^17^. Comparison of protein sequence revealed the lion contig pol and Gag domain to be 60% and 48% identical to both exogenous and endogenous FeLV (which between them displayed 95% and 97% identity in the two domains) (Supplementary Fig. S3).

### Procedure for assessing genetic variation

**Figure 1:**
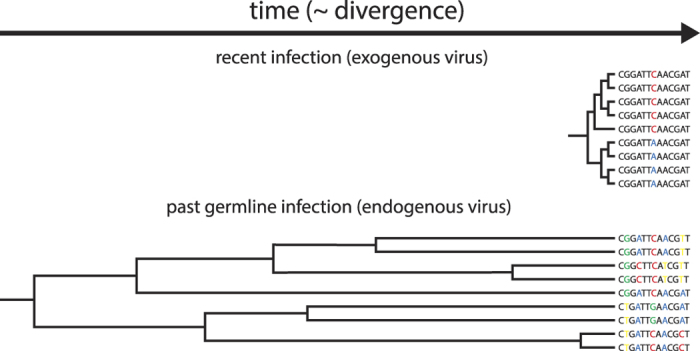
Retrovirus evolution. Schematic depiction of the evolutionary relationship between exogenous (top) and endogenous viruses (bottom). The different evolutionary histories for endogenous and exogenous retroviruses will result in different levels of genetic diversity between individual virus sequences from a sample. Whereas some endogenous retroviruses have resided within genomes for more than 100 million years^38^, individual exogenous retrovirus sequences will all coalesce in the viral sequence that initially infected the individual.

**Figure 2:**
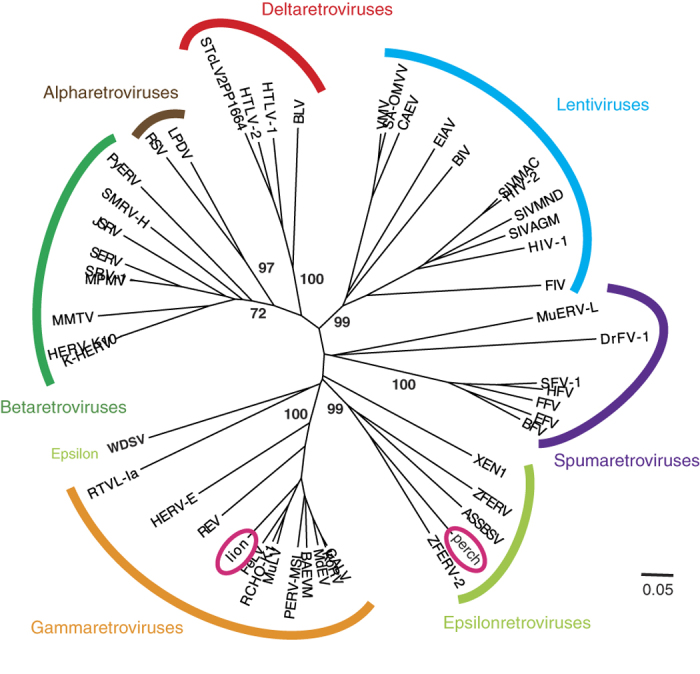
Neighbor-joining tree of integrase protein sequences. The predicted integrase sequences from the novel retroviruses (perch and lion) are aligned against a collection of retrovirus integrase sequences obtained from Gypsy database 2.0^36^. Bootstrap support from a 1000 replicates shown for selected branches. Retrovirus subfamilies are indicated. Bar denotes genetic distance.

**Figure 3:**
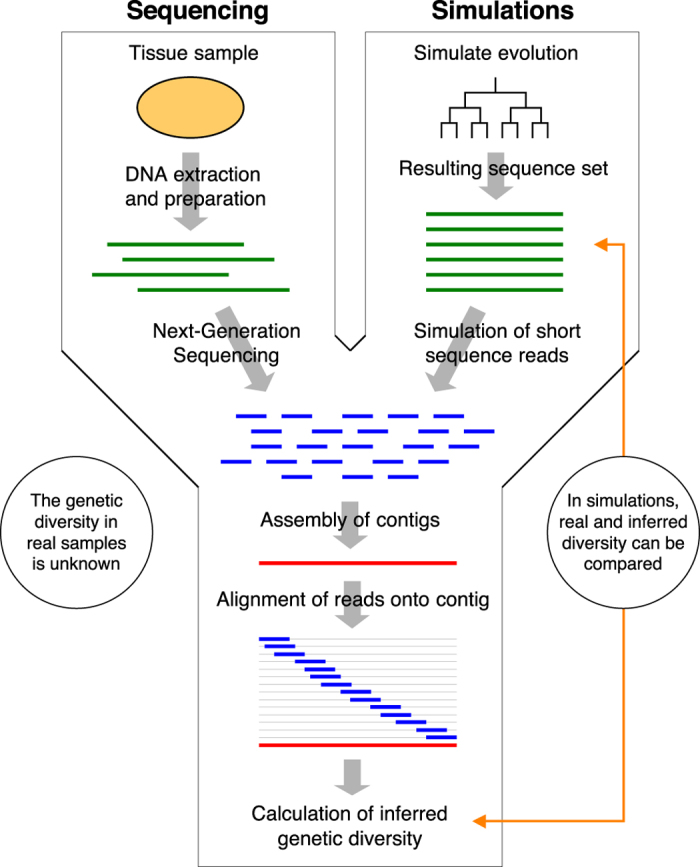
Schematic overview of experimental and analytical procedures. To detect novel viruses (left track, ‘Sequencing’), NGS is applied to DNA templates extracted from tissue samples, and the resulting short sequence reads are assembled into larger contigs. The genetic diversity is assessed by aligning short sequence reads onto the contig sequence, resulting in an alignment in which the number of sequences differs across the alignment. Diversity measures can be applied to this alignment. To establish if this inferred level of genetic diversity reflects the true level present in the DNA templates simulations are performed (right track, ‘Simulations’). Here, the true genetic diversity is known from the parental sequences used to generate the short sequence reads.

To assess if these contigs represent exogenous or endogenous retroviruses, we wished to record the genetic variation between the viral sequences (Fig. 1). However, as the Illumina sequence reads will not represent entire proviruses but rather short fragments derived from a pool of genomic provirus copies, the diversity had to be estimated exclusively from the short sequence reads. Sequence reads were aligned by mapping them back onto the contig sequence. In essence, this corresponds to an alignment with a variable number of sequences across the length of the alignment (Fig. 3). The measures of genetic variation that can be applied to such alignments are limited by the fact that short reads hinder the inference of haplotypes, and we tested the following measures: nucleotide diversity, minor allele frequency (calculated as the combined non-major allele frequency), and the proportion of variable sites. We first needed to establish whether the genetic diversity present in the proviral sequences could be inferred from the short read alignments, and for this we turned to simulations (Fig. 3).

### Inferred genetic variation from short sequence reads

We performed a range of simulations crudely mimicking different evolutionary scenarios. In practice, we took a sequence and duplicated this sequence and its resulting ‘offspring’ with each nucleotide being randomly substituted during duplication with a given probability. Although the starting sequence could have been produced *in silico*, we used the 8735 nucleotides long genome of Human Immunodeficiency Virus (HIV) strain Bx08 simply for convenience. Duplications were repeated for 5 generations resulting in final sets of 32 sequences, and were performed with a range of substitution rates (Table 1). As our aim was to test if genetic variation can be inferred from short sequence reads and not to imitate viral evolution in particular, it should be stressed that these rates are not intended to reflect known viral mutation rates but merely generate a range of sequence divergences. The above was performed with the full-length 8735 nucleotide sequence or with subsets of 5000 or 3000 nucleotides.

From these sequence sets, we simulated the generation of 100 nucleotides long paired-end sequence read (see Methods). Reads were simulated with no sequencing errors and with an error rate of 0.02 per base call. Although the current sequencing error rate of the Illumina platform is considerably higher, post-production filtering and trimming typically renders an effective error rate of 0.02 per base call realistic^18^. To mimic our approach for detecting novel viruses the resulting reads were assembled, the longest assembled contig (if any) was selected, and the read sequences were subsequently mapped to the selected contigs to produce a guided alignment of read sequences (Fig. 3). For all scenarios of substitution rates, sequence sizes, and error rates we simulated sequencing efforts of 100, 1000, and 10000 read pairs. This resulted in 144 different sequence scenarios (Table 1), which were all simulated a 100 times to assess the within-scenario variation.

The measures of genetic variation were applied to the simulated read sequence alignments from above. As measures from simulated sequence reads can be compared to measures from the sequences from which the reads were derived, this allowed us to establish the relationship between true genetic diversity and diversity inferred from short sequence reads aligned onto an assembled contig (Fig. 3).

**Figure 4:**
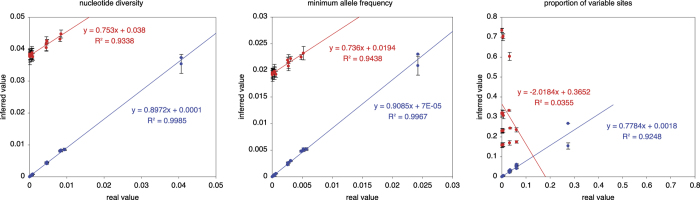
True and inferred genetic variation. Relationship between the true underlying genetic variation (x-axis) and the variation inferred from short sequence reads (y-axis) is shown. Error bars on inferred values are plus/minus one standard deviation obtained from 100 simulations. Blue lines and dots denote simulations with no sequencing errors, red denote simulations with a sequencing error rate of 0.02. Linear regressions (y) and coefficients of determination (R^2^) are shown on the plots.

For simulations with no sequencing errors, all three measures produced results very similar in value to the true genetic variation (Fig. 4, blue lines). However, a major challenge is to distinguish between rare alleles and variant calls resulting from the relative high error rates of NGS technologies. This is clearly reflected by the deviation between values inferred from sequence reads and the true values in simulations with sequencing errors (Fig. 4, red lines). Importantly, although deviating from the real values, both nucleotide diversity and minor allele frequency measures inferred from read sequences are highly correlated with measures from the parental sequences (Fig. 4). We therefore conclude that the relative (albeit not absolute) level of genetic variation can be reliably inferred from short NGS read sequences using nucleotide diversity and minor allele frequency.

### Characterizing genetic diversity in novel retroviral sequences

**Figure 5:**
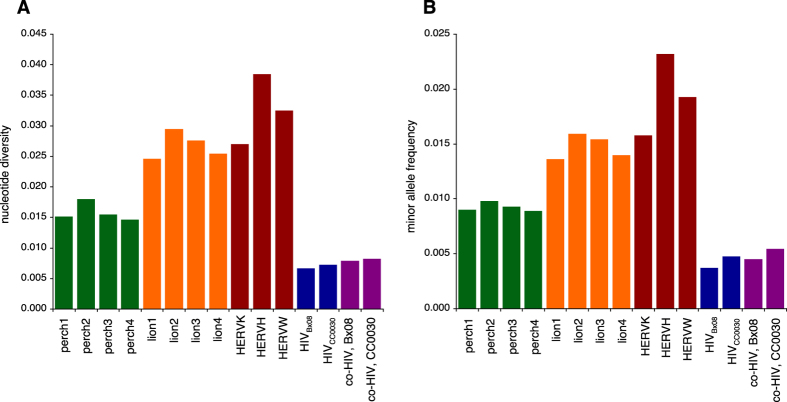
Inferred genetic variation. Bars denote measures of genetic variation inferred from short sequence reads derived from novel retroviruses from perch and lion (perch1–4 and lion1–4, respectively), three known human endogenous retroviruses (HERVK113, HERVH, and HERVW), two exogenous retroviruses (HIV_Bx08_ & HIV_CC0030_), and from HIV samples mixed with both sequence reads from HIV_Bx08_ and HIV_CC0030_ to mimic co-infection. Mixed reads pools were aligned onto the consensus sequence of either Bx08 (co-HIV, Bx08) or CC0030 (co-HIV, CC0030). (**A**) Nucleotide diversity. (**B**) Minor allele frequency.

We next aligned the sequence reads from our lion and perch samples against their corresponding retroviral contigs and calculated the nucleotide diversity as well as the minor allele frequency. To estimate an upper level of genetic variation found in exogenous retroviruses, we obtained DNA sequence reads from *in vitro* cultures with two different strains of HIV, Bx08 and CC0030^19^,^20^. A combination of high mutation rate, short generation time, and recombination makes HIV the retrovirus with the highest level of known intra-host genetic variation^21^,^22^, and thus suitable as a representative exogenous retrovirus against which to compare retroviral sequences of unknown origin. Reads from these two samples were aligned against the HIV reference sequences, and nucleotide diversity and minor allele frequency were calculated. To get variation from known endogenous retroviruses, the DNA sequence reads from the HIV-infected blood cell samples (above) were further aligned against the consensus sequence from the human endogenous retrovirus HERVK113 (GenBank accession AY037928), HERVH (M18048), and HERVW (AF072506), and the procedure was repeated. The relatively recent activity of HERVK113 in the human germ line is witnessed by the presence of polymorphic insertions among human individuals^23^. As expected, using the older human endogenous retroviruses, HERVH and HERVW resulted in higher levels of genetic variation (Fig. 5). The genetic variation between endogenous retrovirus family members will vary depending upon the age and the number of members in the genome, and we would not necessarily expect our retroviruses to display similar levels of genetic variation if they were of endogenous nature. To allow for statistical testing, reads from perch and lion were split into the four samples that they were originally derived from (see Materials and Methods). Both measures of genetic variation for lion and perch retroviruses are significantly higher than the variation found for HIV (Student t-test, Bonferroni-corrected, and assuming equal variance. Nucleotide diversity: perch vs. HIV; p = 5.8 × 10^−3^, lion vs. HIV; p = 1.1 × 10^−3^. Minor allele frequency: perch vs. HIV; p = 1.4 × 10^−3^, lion vs. HIV; p = 1.2 × 10^−3^) (Fig. 5). The retroviral contigs therefore display significantly higher levels of nucleotide diversity than the presumed upper level of diversity within exogenous retroviruses (as represented by HIV), strongly suggesting that the novel retroviruses are of endogenous origin. Another point of interest is the fact that the HIV samples show inferred nucleotide diversity levels of approximately 0.007 (Fig. 5), which is well below the observed range of simulations with a sequencing error rate of 0.02 (Fig. 4), suggesting that our practical error rate is considerably lower than this value.

### On the possibility of co-infection

In theory, infection by multiple closely related exogenous retroviruses could generate an apparent higher level of genetic diversity, thereby mimicking the presence of an endogenous retrovirus. To test this, we mixed roughly equal amounts of sequence reads from the two HIV cultures (app. 10 million reads from each). These reads were then mapped onto either of the HIV consensus sequences, resulting in slightly elevated values of inferred nucleotide diversity and minor allele frequency (Fig. 5), that are still significantly smaller than values from perch and lion sequences (not shown).

The two HIV strains are 81% identical at the nucleotide level with large regional differences in identity, and read coverage is—especially for strain CC0030—very unevenly distributed across the genome (Supplementary Fig. S4). If reads from the two HIV strains are predominantly mapping to regions of high similarity between the two genomes, this is not expected to produce higher levels of inferred nucleotide diversity. Hence, our test case for co-infection may not exhaustively exploit the effect that co-infection may have on inferred genetic diversity. We expect the highest impact on inferred genetic diversity would result from cases of co-infection where divergence between infecting strains is distributed across the genomes and where sequence copies are present in similar numbers. To assess the effect of such scenarios, we again turned to simulations.

### Simulations of co-infection scenarios

**Figure 6:**
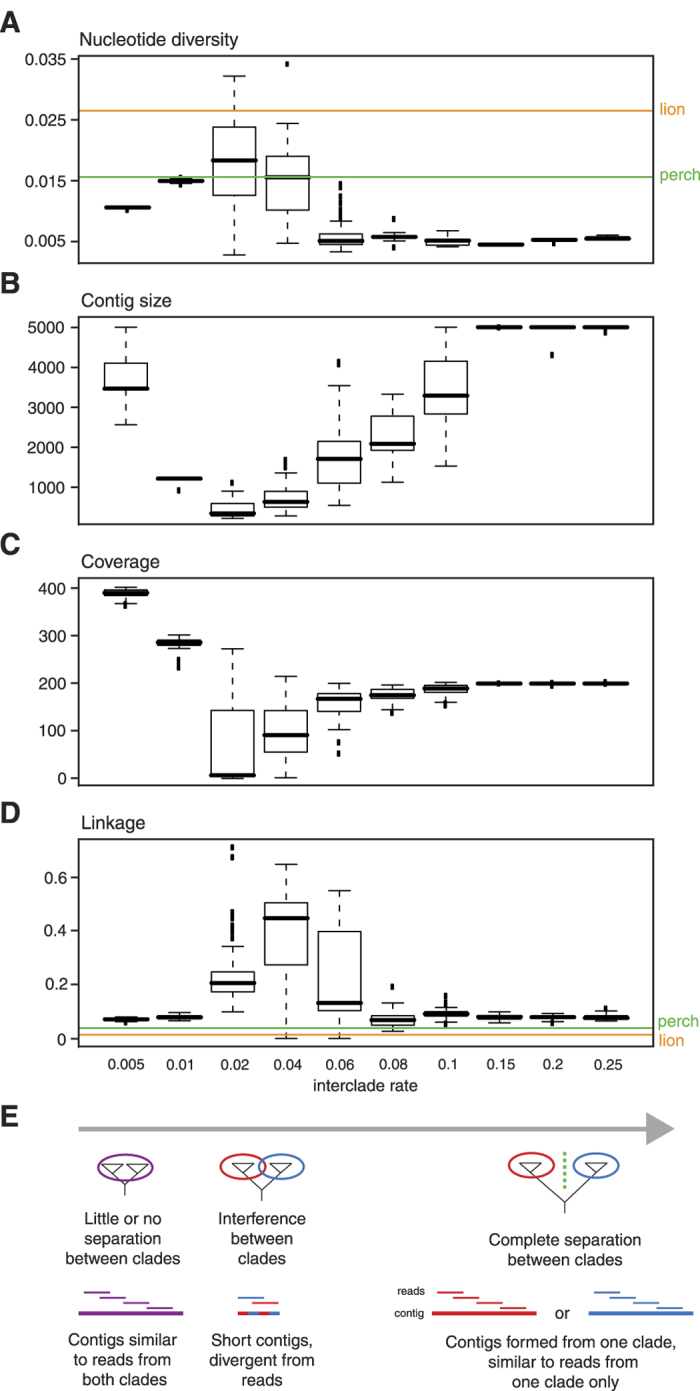
Simulation of co-infection scenarios. Box plots of nucleotide diversity (**A**) contig sizes (**B**) read coverage (**C**) and linkage (**D**) is shown for different levels of interclade rates (values shown below linkage plot). Bars, boxes, whiskers and squares show median, 1st and 3rd quartile, 1.5 times the interquartile range, and outliers, respectively. In the plots of nucleotide diversity and linkage, the corresponding values observed for the perch and lion retroviral contigs are indicated by horizontal lines in green and orange, respectively. Below the plots, the hypothesised effects of different interclade rates are shown (**E**).

In principle, we would repeat our simulations of sequences being duplicated for 5 generations resulting in final sets of 32 sequences. But to imitate sequences from two related exogenous viruses, we now vary the substitution rate at the first duplication round (corresponding to the evolutionary distance between two virus strains) and keep the subsequent substitution rates fixed (the evolutionary distance between individual members of the infection). This will result in two clades of closely related sequences, and we refer to the substitution rates at the first duplication rate is the interclade rate, and the subsequent substitution rate as the intraclade rate. The intraclade rate was fixed so that sequence diversity within clades was similar to what the inferred from the HIV samples above, whereas different interclade rates in the range of 0.005 to 0.25 substitutions/site/generation were simulated. All simulations were done using a sequence size of 5000 bp and 10000 sequence reads (100 bp paired-end). As before, simulated sequence reads were assembled into contigs, and read sequences were aligned back onto the contigs. For these new simulations, we recorded the inferred nucleotide diversity, read coverage, and size of assembled contigs (Fig. 6).

### Contig assembly and coverage in co-infection scenarios

For interclade rates in the range of 0.01–0.04 we see that inferred nucleotide diversity levels do indeed reach levels exceeding the observed values for the perch and lion retroviruses (Fig. 6A). Other noteworthy observations are that intermediate interclade rates produce short contigs (Fig. 6B) with low coverage (Fig. 6C), and that high interclade rates display coverage of around half that observed for the lowest interclade rate (Fig. 6C).

The hypothesized reasons underlying these observations are outlined in Fig. 6E: For high interclade rates, the reads from the two clades are so divergent that the longest contig will exclusively be formed from reads derived form one of the clades. By comparing the contig sequences to the parental sequences, we can see that this is actually the case (Supplementary Fig. S5). In contrast, for the lowest interclade rate reads from the two clades are similar enough for contigs to be formed from reads derived from both clades. This is consistent with the observation that the coverage at the interclade rate of 0.05 is roughly twice that of the highest interclade rates (Fig. 6C). In between these extremes, the similarity of reads between the two clades interferes with the assembly step, producing very short contigs with low coverage (Fig. 6B,C). Hence, it appears that the very conditions that in which the similarity between the two infecting viruses is high enough to influence the inferred nucleotide diversity are the same conditions that impair the assembly of contigs from the two pools of read sequences. This is reassuring, as the probability of obtaining retroviral sequence contigs as large as those of perch and lion is then minimized in cases of co-infection with two exogenous retroviruses.

### Linkage analysis

Still, one cannot rule out the occasional assembly of a contig from a co-infection with two exogenous retroviruses that, although short and with relatively low coverage, will display an inferred nucleotide diversity resembling that of an endogenous retrovirus. We therefore sought additional markers to distinguish between an endogenous retrovirus and co-infection with exogenous retroviruses.

In a co-infection scenario, one would expect a high degree of linkage between substitutions, as a large proportion of substitutions will have taken place in the branches leading to the two clades. As no such degree of linkage is expected for an endogenous retrovirus, this could potentially differentiate an endogenous retrovirus from co-infection with two exogenous retroviruses. To test this, we recorded the level of linkage between substitutions (as defined by base differences observed between sequence reads and the consensus contig) in our simulations of co-infection. As seen from Fig. 6D, elevated levels of linkage is indeed observed for the interclade rates for which consensus contigs are derived from both clades. The maximum number of simulations showing linkage values smaller or equal to the actual level observed for the perch and lion contig (0.039 and 0.014, respectively, Fig. 6D), is three for the perch (in the interclade rate of 0.04) and 1 for the lion (in the interclade rates of 0.04 and 0.06). This suggests that observed levels of linkage are lower than simulated values at a level of significance not exceeding 0.03 for perch and 0.01 for lion (i.e. 3 and 1 simulations out of 100, respectively).

Based on the above, we again conclude that the perch and lion retroviruses are of endogenous origin, and that the contigs are not produced from co-infection of two exogenous retroviruses.

### Analysis of lion reference genome

For the lion retrovirus, further support can be found in the recently published genomes of African lion and white African lion^24^. Ample sequences highly similar to our retroviral lion contig were found in both lion genomes (Supplementary Fig. S6), and if ignoring the unlikely possibility that all sampled lions are infected by a similar exogenous retrovirus, this strongly supports the conclusion of the retrovirus being of endogenous origin.

## Conclusions

Throughout evolution, a broad range of retroviruses have repeatedly been integrating in vertebrate genomes^25^. We here present two novel retroviral sequences from lion and pike-perch. From simulations, we show that the level of genetic diversity can reliably be inferred from short sequence reads. The inferred nucleotide diversity levels of the novel retroviral sequences are significantly higher than levels observed for HIV infections, which represent the exogenous retrovirus with the highest known levels of intra-patient genetic diversity.

Further simulations of co-infection scenarios suggest that this may under certain circumstances result in higher levels of inferred genetic diversity, although these specific circumstances also impair the assembly of contigs. By analysing the level of linkage in the sequence reads underlying our retroviral contigs, we rule out the possibility that these are produced from two co-infecting retroviruses.

One limitation of our approach is that very recent endogenous retroviruses will not have accumulated enough genetic changes to be identified as endogenous. This will obviously exclude the analysis of retroviruses such as KoRV that exists as both an endogenous and an exogenous virus in koala populations^26^,^27^,^28^. Nevertheless, the presented analysis provides a quick means for the initial assessment of retroviral sequences that does not require further labour- and time-intensive lab work or sampling.

In summary, we conclude that the lion gammaretroviruses and the pike-perch epsilonretroviruses are both of endogenous origin. We suggest that by analyzing viral sequencing in an evolutionary perspective, endogenous retroviruses of ancient origin can be distinguished from exogenous retroviruses, and show that measures of genetic variation can be applied to sequence reads produced from NGS technology to make the distinction possible.

## Methods

### Samples

Samples were obtained from the brain and liver of two clinically healthy euthanized lions at Copenhagen Zoo, and from lymphosarcomas in the skin of four pike-perch specimens collected at a Danish fish farm, which had been sent for diagnostic purposes. None of the animals were killed for the purpose of this study.

### Virion enrichment, nucleic acid extraction and library build

Fresh frozen samples were thawed and transferred to 400 μl cold PBS. Two stainless steel beads of 2–3 mm diameter were added to the samples, which were subsequently homogenised using the TissueLyser II (Qiagen) for 6 minutes at 30 Hz. Homogenates were centrifuged for 2 minutes at 1400 g to remove tissue debris, and the supernatants were subsequently filtered through 5 μm centrifuge filters (Millipore). The filtrates were nuclease treated to remove unprotected nucleic acids using 14 μl TURBO DNase (2 U/μl) (Ambion), 12 μl Baseline-ZERO DNase (1 U/μl) (Epicentre), 16 μl RNase Cocktail Enzyme Mix (Ambion), and 40 μl 10 × TURBO DNase buffer in a total volume of 400 μl, and incubated at 37 °C for two hours. Nucleic acids from the virion-enriched samples were extracted using the High Pure Viral RNA Kit (Roche) according to the manufacturers instructions, with the addition of 10 μg linear acrylamide carrier (Applied Biosystems).

Sequencing libraries were built on the DNA fraction, which includes enriched DNA viruses and leftover host DNA, using the Nextera XT DNA Sample Preparation Kit (Illumina), according to the manufacturers instructions. Briefly, DNA was fragmented and tagged with adapters during a 5-minute incubation at 55°C with Nextera Tagment DNA Enzyme containing a modified transposase enzyme complexed with adapters. The tagmented DNA was amplified by 12 cycles of PCR, during which index sequences and sequencing adaptors were added to the DNA libraries. The amplified, indexed DNA libraries were purified using the Agencourt AMPure XP PCR Purification system (Beckman Coulter). 100 bp paired end sequencing was performed on the Illumina HiSeq 2000 platform. Analysed read sequences are deposited at the European Nucleotide Archive (http://www.ebi.ac.uk/ena), accession PRJEB10284.

### Sequence analysis

Adapter sequences were removed with AdapterRemoval^29^, and remaining read sequences were assembled using Ray Méta^30^. Initial detection of retroviral sequence relied on mapping of contigs to several databases from EBI (http://www.ebi.ac.uk/) consisting of the genomes of the archaea, archaea-viruses, bacteria, phages and viruses databases, and to fungi and protist genomes from NCBI (http://www.ncbi.nlm.nih.gov/). This was done using PROmer from the MUMmer package^31^. Reads were subsequently mapped back onto the resulting retroviral contigs using bwa^32^ version 0.6.2 with default parameters to produce a guided alignment of the read sequences. This allows for an edit distance of 0.04, meaning 4 nucleotide mismatches for a 100 nucleotides long sequence read. Measures of genetic diversity were calculated from pileup files produced using samtools^33^. From the pileup files, only positions with a minimum coverage of 5 reads were used for calculating nucleotide diversity, minor allele frequency, and proportion of variable sites. Overall coverage and number of positions with the minimum coverage are provided in Supplementary Table S1. The consensus sequence itself was omitted from analysis. Nucleotide diversity calculated per position as suggested in reference^34^ and generalized in a blog post at: http://binhe.org/2011/12/29/calculate-nucleotide-diversity-per-base-pair-summation-method/. For an alignment with N positions, with alleles S = [A, C, G, T], nucleotide diversity is calculated as:


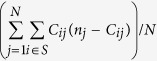


where *C*_*ij*_ is the count of allele *i* at position *j*, and *n*_*j*_ is the total count of alleles at position *j*. Minor allele frequency was defined as the combined frequency of all non-major alleles and averaged across all positions. Proportion of variable sites was simply calculated as all positions with multiple alleles divided by the total number of positions. Simulated short sequences were generated with wgsim (https://github.com/lh3/wgsim). All sequences were simulated as 100 bp paired-end sequences with a total insert size of 500 bp (i.e. 300 bp between the two read sequences). Assembly, alignments and diversity calculations were carried out as for the real data.

### Phylogenetic analysis

Protein domains were identified using pfam^35^. Protein sequences from a range of retroviruses was downloaded from Gypsy Database 2.0^36^. Alignments and neighbor-joining trees were constructed using clustal^37^, and trees were manipulated in FigTree (http://tree.bio.ed.ac.uk/software/figtree/).

### Linkage analysis

For a given sample or simulation, all substitutions and their coordinates in the contig sequence were recorded from the pileup files. For each read sequence being mapped onto the contig, the potential substitutions being covered by that read was determined from the mapping coordinates. For each pair-wise combination of covered potential substitutions, the occurrence of one or both substitutions was recorded. This was cumulated for all sequence reads, the linkage between substitution A and B was calculated as (occurrences of substitution A and B)/(occurrences of substitution A only + occurrences of substitution B only + occurrences of substitution A and B), and the average linkage for all substitution pairs was calculated.

### Lion genome analysis

Sequence read data from African lion (SRR836361) and white African lion (SRR836370)^24^ were downloaded from Sequence Read Archive at NCBI (http://www.ncbi.nlm.nih.gov/sra). A subset of 100 M read pairs from each sample was mapped onto the lion retrovirus contig using bwa^32^.

## Additional Information

**How to cite this article**: Mourier, T. *et al.* Characterizing novel endogenous retroviruses from genetic variation inferred from short sequence reads. *Sci. Rep.* **5**, 15644; doi: 10.1038/srep15644 (2015).

## Supplementary Material

Supplementary Information

## Figures and Tables

**Table 1:** Parameters used for simulations of NGS data sets.

Substitution rates (site^−1^ × generation^−1^)
0
1 × 10^−5^
5 × 10^−5^
1 × 10^−4^
5 × 10^−4^
1 × 10^−3^
5 × 10^−3^
1 × 10^−2^
Size of original sequences (nucleotides)
3000
5000
8735
Error rate (base call^−1^)
0
0.02
Simulated reads (number)
100
1000
10000

All combinations (8 substitution rates × 3 sequence sizes × 2 error rates × 3 read numbers, 144 in total) were performed with 100 iterations.
