# Supplementary material for: Characterizing novel endogenous retroviruses from genetic variation inferred from short sequence reads
Source: Sci Rep. 2015 Oct 23;5:15644. doi: 10.1038/srep15644 (PMC4616055; doi:10.1038/srep15644)
Supplement: Supplementary Information [file srep15644-s1.pdf]

Supplementary Materials

Characterizing novel endogenous retroviruses from genetic variation inferred from short sequence reads

Tobias Mourier, Sarah Mollerup, Lasse Vinner, Thomas Arn Hansen, Kristín Rós Kjartansdóttir, Tobias Guldberg Frøslev, Torsten Snogdal Boutrup, Lars Peter Nielsen, Eske Willerslev, Anders J. Hansen

Legends to supplementary figures

Supplementary Figure S1. Sequences of identified retroviral contigs. Protein domains identified at the amino acid level by pfam are highlighted in colours according to legend above sequences. Sequences are shown as reverse complements relative to contigs produced by Ray Méta.

Supplementary Figure S2. Alignment between amino acid sequences (RNaseH and integrase) from fish retroviral contig and 3 closely related fish retroviruses obtained from Gypsy database.

Supplementary Figure S3. Alignment between amino acid sequences (gag and pol) from lion retroviral contig and 3 closely related retroviruses obtained from Gypsy database.

Supplementary Figure S4. Coverage and identity between two HIV strains. Read coverage is from two in vitro cultures of human cells infected with HIV strain Bx08 and CC0030, respectively (Vinner et al., submitted). Identity is the fraction of identical positions in an alignment between the two HIV genome sequences. All values shown as averages across non-overlapping 100 base pairs windows.

Supplementary Figure S5. Plot showing the identities between simulated contigs and parental sequences. From the co-infection simulations, a consensus sequence was made from each clade of sequences. The longest contigs was aligned to both consensus sequences and the overall sequence identity was recorded. The identity to the consensus sequence to which the contig had the highest identity was recorded as 'best', and the lowest as 'worst' The 3 plots shown the same values, but with different resolution on the y-axes. The values obtained from different interclade rate scenarios are shown on the y-axis with values for 'best' and 'worst' identity grouped as 'B' and 'W'

Supplementary Figure S6. Read sequences reads from African lion (SRR836361) and white African lion (SRR836370) mapped to the lion retroviral contig. 100 M reads were used from each lion genome.

Figure S1

Protein domains

pfam ID	description
Gag_MA	Matrix protein (MA)
Gag_p30	Gag P30 core shell protein
RVP	Retroviral aspartyl protease
RVT_1	Reverse transcriptase (RNA-dependent DNA polymerase)
RNase_H	RNase H
rve	Integrase core domain

```
>lion
GTGGTCTTTGGGGATCACGAATTCTGGGCATAACATTTGGGGGCTCGTCCGGGATCCCCAAGACCCCTGAGGGACCCCCGACCCGGAGAGCCTGACTGGCCACGGTTAGTGTCTGTTTGT
CCTGACTGGCCACGGTTAGTGTCTGTTTGTTTGTTCTGTCTTTTCTGTGTGAGCTCACTTCTGGAGTTCTGGTAGTGCCCGACGCGGTCTAAGTGGACGCACTGGAGGACCACGGGCCGG
AAGTGGACGCACTGGAGGACCACGGGCCGGGAGTTTCGGAAGACGTTCCGATTCTCCCTTCTGGAGGGACGTGGAATCCCCTCAAAGGTCTGAGACGAGGCGGGTCGCTCCCGCTGGTCG
TGAGACGAGGCGGGTCGCTCCCGCTGGTCGGRGTGAGGCCGTCGTCTTTGGAGGGACGTGGAATCCCCTCATCGGTTTTGGAGGGACGTGGAATCCCCTCAAATGTCTAAGCTAGCTTTC
GAATCCCCTCAAATGTCTAAGCTAGCTTTCAGTMTTGCTTCCATGGAGTTGGAAGATTTTCTAGGGGCCCTCTGTTTGTCTGTTTTTGTGTTTCTCTGTTTTGTTCTGTGGACTTACTGG
TTTCTCTGTTTTGTTCTGTGGACTTACTGGACGGACGTTATGGGACAGACTCAGACTACTCCTCTAAGTATTATGATTGATCACTTTAAGGATGTGAGGGGAAGAGCTAACAACCTCAGT
GATGTGAGGGGAAGAGCTAACAACCTCAGTGTGGAAGTCCGAAAGGGTCGGTGGCAGTTTTTTTGTTCTAGCGAGTGGCCAACTTTCAATGTCGGATGGCCACCAGAGGGGACCTTCGAC
GTCGGATGGCCACCAGAGGGGACCTTCGACCTCCCTACCATCCACCGAGTCAGGAGTATCATCTCTCAGCCTAAGACGGGCCATCTTGATCAGCTCCCTTACATTATCACTTGGCAGGAC
CAGCTCCCTTACATTATCACTTGGCAGGACCTTGTGGAAGACCCACCCTCTTGGCTTAAACCCTTCCTAGCCCCGCTCCCTCCGGAGCCAAAACCCATTCTTGCTTTGCAGGGGACAAAG
AAACCCATTCTTGCTTTGCAGGGGACAAAGAAGAAGAAAAGTCTTATCCAGCCTTCAGCACCCCTCTACCCTGTCTTACAGGGGGGTACTGAAGAAGAATTAATTTTTCCTCCCTCGTAT
GAAGAAGAATTAATTTTTCCTCCCTCGTATAACCCCTCTAGGATGCTGGAAGAACACCATCCTCCCCCTCCGGGGGAGGCAGATGCTGTTCCGAGAGCGGGAGGCGGAAACGCTCCAGTG
CCGAGAGCGGGAGGCGGAAACGCTCCAGTGGGAAGCCCGCCCTTCACCAGACAAAGGGCTCAGAGGGAGCAATCCGCCTCCGCCGCCGACTCCACTATTCTGCCCCTGCGAGCCACCGGA
TCCACTATTCTGCCCCTGCGAGCCACCGGACCCCCAGACGCGGAGGGGAACCAGCCCCATCACTATTGGCCTTTCGCCACTAGTGACCTCTACAATTGGAAAGCTCAGAATCCTAAGTTT
TACAATTGGAAAGCTCAGAATCCTAAGTTTTCCGAGAAACCGGCAGGGCTTATTGATTTATTAGACTCTGTTCTTTTTACCCATCAGCCCACGTGGGACGATTGCCAGCAGCTTTTGCAG
ACGTGGGACGATTGCCAGCAGCTTTTGCAGGTCCTGTTCACGACTGAAGAAAGAGAAAGAATCGTCAACGAGGCCCGAAAACTAGTTCCGGGCACAGACGGGAATCCCACCACCAACCAG
GGCACAGACGGGAATCCCACCACCAACCAGGCTCAGATAGATGCCTCCTTCCCCTTAACTCGGCCCCAGTGGGATTTCAACACGGCAGAAGGTAAGGAGAGGCTCCGGGTCTACCGCCAG
GGTAAGGAGAGGCTCCGGGTCTACCGCCAGACTCTAATGGGGGGTCTCCGAATGGCTGCTAGAAAGCCAACCAATTTGGCCAAGGTAGGAAATGTACAACAGGGAAAAGATGAATCTCCG
AATGTACAACAGGGAAAAGATGAATCTCCGGCTGCCTTTTTAGAACGGATCATGGAGGCATTCCGTACCTATACCCCCATGGATCCAGAGGCTCCGGAAAGCAAGGCAGCTGTTATCATG
GCTCCGGAAAGCAAGGCAGCTGTTATCATGGCCTTTGTAAACCAATCGGCCATAGACATTAGGAGAAAATTACAGAAAATAGATAGACTAGGAGAAAAAAGTCTGCAGGACTTACTGGTG
GGAGAAAAAAGTCTGCAGGACTTACTGGTGGTAGCCGAAAAGGTATATAATAACCGGGAGCTTCCTGAGGACAAGCAGGCTCGCGCCATGGCGGCTGCCAGCAGTAAGCAGACTCGAGAC
GCGGCTGCCAGCAGTAAGCAGACTCGAGACCTGGCCAGAATACTACTAGCTACCACTGCTGACTCCCCTGAGGAACGAGACCGCCGTCTCTGGCAGCTGGCAGACGACGCAAGAAAAGGT
TGGCAGCTGGCAGACGACGCAAGAAAAGGTAAAAGAACCACCAAGGGGGGGAAGCAGAGGCTGCAGAAGGATCAGTGCGCATACTGCAAGGAGATAGGGCATTGGGCCCGAGATTGTCTG
GAGATAGGGCATTGGGCCCGAGATTGTCTGAAAAGGGCCGGCGGGAAAGGAAGCAAGACTGATCGAGTAAAAGTCCTAGAGCTAGATGAACTAAGTGATTAGGGGAGTCGGGGTTCGGAC
CTAAGTGATTAGGGGAGTCGGGGTTCGGACCCTCTCCCCGAACCCAGGGTAACTCTTAAAGTGGAGGGGACCCCTGTTGACTTCCTTGTCGACACCGGAGCACAACATTCGGTCCTCCGC
GACACCGGAGCACAACATTCGGTCCTCCGCACCCCACAAGGAAAACTAGCCAGCAAGAAGTCCTGGGTACAAGGGGCAACTGGTATGAGCCAGTATTCATGGACTACCCGAAGAACAGTA
CAGTATTCATGGACTACCCGAAGAACAGTAGATTTGGGAACGGGCCGGGTATCCCACTCCTTTATGGTAATACCAGAATGCCCCTACCCGCTGTTAGGACGGGACTTACTGACCAAGATT
CTGTTAGGACGGGACTTACTGACCAAGATTGGAGCTCAGATAACTTTCAGACAAGGGGGGCCTCAGGTCACCGATGGCAAGGGCCACCCCATCCAGGTCCTGACCATGAAACTGGAGGAT
ATCCAGGTCCTGACCATGAAACTGGAGGATGAATACCTCCTCCACCAGGAGGCGCTCCCGAGAGAGGATAATATAGACAGATGGCTACAAGAATTCCCCTCGGTTTGGGCAGAGACTGGG
GAATTCCCCTCGGTTTGGGCAGAGACTGGGGGGGGGGATGGGACTAGCCGCTCATAGGACCCCAGTCCTGGTAGAGCTCAAGCCAGGAGAGAGTCCGGTAAGGATCAAACAATACCCCATG
AGTCCGGTAAGGATCAAACAATACCCCATGTCACAGGAGGCCCGGAAGGGGATCCAGCCACACATCCGGAGACTACGAAGCCTAGGGGTACTAGTTCCTTGCCAGTCTGCCTGGAACACC
CTAGTTCCTTGCCAGTCTGCCTGGAACACCCCCTTACTGCCGGTCAAAAAGCCTCACACAAATGACTACCGACCGGTACAAGACCTCCGGGAAGTAAATAAGAGGGTCGCGGACATACAC
GAAGTAAATAAGAGGGTCGCGGACATACACCCAACTGTTCCCAACCCATATACTCTCTTGAGCTCCTTGGCGCCCTCCAGGGTCTGGTATACTGTACTAGATTTAAAGGACGCCTTCTTC
ACTGTACTAGATTTAAAGGACGCCTTCTTCAGTCTGCCGCTGGCACCCCAGAGCCAACCCCTGTTCGCCTTCGAGTGGCATGATCCGGAGGAGGGCTACAGTGGGCAACTCACCTGGACA
GAGGGCTACAGTGGGCAACTCACCTGGACACGGCTACCTCAGGGATTCAAAAATTCACCCACCATCTTCGACGAGGCACTACACGAGGACCTGGGTGAGTACAGAAGGGAGCACCCTGGC
CTGGGTGAGTACAGAAGGGAGCACCCTGGCCTCACCCTCCTACAGTACGTAGATGACATCCTGATTGCTGCTGACACGGCCAAAGACTGTGAGCGAGGGACCCAGGACCTGCTGGCTACC
GAGCGAGGGACCCAGGACCTGCTGGCTACCCTGGGGGCCTTAGGGTACCGGGCATCCGCGAAGAAGGCTCAGATATGCAGGGAGAGGGTAAGTTACCTGGGATATATCCTGGAGGGCGGA
```

Figure S1

AGTTACCTGGGATATATCCTGGAGGGCGGACAGCGGCGGTTATCAGATGCCAGAAAAGAAACTGTCCTAAAGATCCCTACTCCCACCTCCCGAAGAGAAGTGAGGGAATTCCTAGGATCA
CGAAGAGAAGTGAGGGAATTCCTAGGATCAGCCGGCTACTGCCGCCTCTGGGTTCCAGGTTTTGCTGAGATCGCCAGGCCCCTATATGAAGCTACCAAAGAGGGGAAAACATTTAAATGG
GCTACCAAAGAGGGGAAAACATTTAAATGGACTGAAAAAGAAGAAATTGCCTTTAATCAGTTAAAAAAGGCCCTCCTAAGTGCCCCAGCCCTGGGCCTACCAGACATTATGAAACCCTTC
CTGGGCCTACCAGACATTATGAAACCCTTCCACCTCTTTGTAGACGAACATAAGGGAATAGCAAAAGGGGTTCTAACTCAAGCCTTAGGCCCCTGGAACCGCCCAGTGGCTTACCTGTCT
CCCTGGAACCGCCCAGTGGCTTACCTGTCTAAGAAACTAGACCCAGTGGCTGCCGGCTGGCCGCCATGCCTAAGAATTATTGCGGCGACAGCACTCCTAGTCAAGGATGCAGACAAACTG
GCACTCCTAGTCAAGGATGCAGACAAACTGACCCTAGGACAGGAGATCTGGATCACAACCCCACACGCCATTGAAGGGGTCCTGAAACAGCCTCCTGATAGATGGATGAGCAATACACGT
CCTCCTGATAGATGGATGAGCAATACACGTGTGACTCATTACCAGAGCCTCCTACTCAACCCTCCACGAGTGCGGTTCCACCCCAGTGCAGCCCTCAATCCTGCAACCCTGCTGCCCGAC
GCCCTCAATCCTGCAACCCTGCTGCCCGACCCTGACCTAGGTGCTCCACTACATGACTGTGCGGGAATCCTGGAACAAGTACATGGATTCCGGATGGACCTGACCGACCAGCCCCTCCCC
CGGATGGACCTGACCGACCAGCCCCTCCCCGATGCCGAGGCTACTTGGTTCACTGATGGCAGCAGCTTTGTGCGAGATGGACACAGGTATGCGGGTGCAGCGGTGGTCACCGAAATGGAC
GCGGGTGCAGCGGTGGTCACCGAAATGGACACCGTATGGGCGGAGGCTCTACCCTCCGGAACGTCAGCCCAGCGAGCGGAGCTCATAGCCCTCACCAAGGCGCTGATGCTGGGAGCTGGA
CTCACCAAGGCGCTGATGCTGGGAGCTGGAAAACGGCTTAACATCTACACAGACAGCCGTTATGCATTTGCCACAGCTCATATTCATGGGGCAATTTATCAGGAGAGGGGGTTACTGACG
GCAATTTATCAGGAGAGGGGGTTACTGACGGCAGAAGGACGGACTATAAAAAATAAGCAGGAGATACTTAACCTGCTTACGGCCTTATGGCTTCCTGCCAAGCTAGCCATTATCCACTGC
CTTCCTGCCAAGCTAGCCATTATCCACTGCCAAGGGCACCAAAAAGCTGATAACCCAGTAGCTAGAGGTAATCGAAAGGCTGACCAGGCAGCCAAGGCAGTAGCCCTTACTTCAGTCCCC
GCCAAGGCAGTAGCCCTTACTTCAGTCCCCACCATGACCATACAACTACCGGACCCGGGAGACCCAGTTTTACCAGACCAGCCCAAATACTCCCAGGAGGAGTTACAGCGGATCAAGAAA
TCCCAGGAGGAGTTACAGCGGATCAAGAAACTCCCCATGGCCCAGGAGATAAAGGGATGGTGGTATACACCTAACAAGGAGCTCGTGCTGCCAGACCGGCTCGGAGTCTCAATATTAGAG
CCAGACCGGCTCGGAGTCTCAATATTAGAGCACATGCATCGGTCTACTCACATGGGGGCCCGAAAATTAAAAGACTTAATCCGACATGCCGGAATCAAGATTCACCAACAGGACACCAAA
GGAATCAAGATTCACCAACAGGACACCAAAATAGAGCAAGTTGTATCTGCCTGCAAGACCTGCCAACTCACCAACGCGAAAGCCACATCAAATAAAAAAGGAACCAGGCTCAGAGGCACC
AATAAAAAAGGAACCAGGCTCAGAGGCACCAGACCGGGAGCCCAATGGGAAGTCGACTTCACTGAAGTCAAACCAGGAAAGTATGGTTATAAATATCTTTTAGTATTTACAGACACCTTC
AAATATCTTTTAGTATTTACAGACACCTTCTCTGGCTGGGTGGAGGCATACCCAACCAAGCATGAAACGGCTCAGACGGTGGCTAAGAAGCTACTAGAAGACATCTTACCCAGGTATGGT
CTACTAGAAGACATCTTACCCAGGTATGGTTTCCTGCCATGGTAGGATCAGACAATGGACCAGCTTTTATCTCTCAGGTAACACAGGCAGTAGCCAAGGCGGTGGGGGCAAACTGGAAA
GTAGCCAAGGCGGTGGGGGCAAACTGGAAATTACATTGTGCTTATAGGCCCCAGAGCTCAGGACAGGTAGAAAGAATGAATAGAACCCTAAAAGAGACCCTTACCAAATTAACCATGGAG
AAAGAGACCCTTACCAAATTAACCATGGAGACTGGCGGGGACTGGGTGACTCTCCTACCGTACGCCCTTTACCGGGTTAGAAACACTCCTTACACTCTGGGTTTTACTCCCTACGAGATC
TACACTCTGGGTTTTACTCCCTACGAGATCATGTTTGGCAGGCCACCCCCTGTTATTCCCAGCCTTCGAGCTGAACTTCTTGCAGAGTTTAAAGATCAAGAACTTTTTCTTTCCTTGAGA
AAAGATCAAGAACTTTTTCTTTCCTTGAGAGGGCTCCAGAGGGCGCACGAGGACATTTGGCCGCGCCTCCGTGCCATCTACGAGGCTGGCCCGACCCCGACACCTCATCAGTACAAGCCG
CCGACCCCGACACCTCATCAGTACAAGCCGGGAGACTGGGTCTATGTCAAGAGGCACCACCGAGAGACTCTCGAGCCGCGCTGGAAGGGACCCTACATTGTAGTGCTGACAACCCCCACC
CCCTACATTGTAGTGCTGACAACCCCCACCGCTCTCAAAGTAGACGGCATCGCGACCTGGGTCCATCACACCCACGTTCGGCCAGCGGACCCCTCCTCGATCCGGAAGGACTTCGTCACG
CCCTCCTCGATCCGGAAGGACTTCGTCACGCGATGGGCCATCAGTCGGGACCAACACAACCCGCTCAAGCTCAAGCTCAAGCTACAGCACATTCGACCCACCTAATATTGGTAACTCTGT
ATTCGACCCACCTAATATTGGTAACTCTGTTAACTCTGCTTGTCATTGCTCATGCTACCGGGAGTCCCCACACCCCCCAAAACATCACCTGGCAGATCATAGATACCAGCTCAGGGACAA
GGCAGATCATAGATACCAGCTCAGGGACAATACTTAATTAGACCTCCCAGAGCCACCCCAGGGACACATGCTTCCCAGAACTTAGCGTAAACTTCCAAACTCTGTTCCCCTTGTCACCAT
ACTTCCAAACTCTGTTCCCCTTGTCACCATAAATGCAGCCGGTCCCATGATTGGGTCCCTAAGCTACATGACAAGGGGGGAATGAAAGGGCGGGCCTATGCCGGCCGACTCCATCTTGTT
CGGGCCTATGCCGGCCGACTCCATCTTGTTCTGTGT

>fish
GGGATGAACTCTCAACCTATTTCTTACTATTCTACTGCTTATTCAGACGTAGAATTAGGGTTACCACCGTGTTATAGAGCAATGGTAGGAGTCTCTTTAATGTATGATAAAGCATCATCA
GTCTCTTTAATGTATGATAAAGCATCATCAGTCACGATGGGTTATCCAATAACAATTCTTACCCATCATAGRCTCAGAAATCTTCTGAACTATGGAAAATATACATTGACTATGCCTAGG
TATGGAAAATATACATTGACTATGCCTAGGCTTAGAGACTATCATAGGCTTCTAGAGCAGGAAGATGTCACCCTGGCAAGGTGTGCCACGGTGAATCCAGCTGAGAATTTGCCAACCCCA
GTGAATCCAGCTGAGAATTTGCCAACCCCAGAGGATGGTGAACAACATGATTGTGTTCAGGAAGCAGAGAAATATTCGAGGCTTAGATCAGATTTACAAGCCCTTCCATTACGTGAGGCA
GATTTACAAGCCCTTCCATTACGTGAGGCAGACCTGGAGTATTGGACTGATGGGTCATGTTATCGTGTGGGAGATAAATTGAGTGCTGGCTATGCAGTAGTAAAAGCACAAGGAACTGGA
TATGCAGTAGTAAAAGCACAAGGAACTGGATTTGTTGTTGAAAAGGCTGAAGTAATACCACAGCCTGCGTCGGCGCAACTTGCAGAACTTGTAGGGTTAACAGAAGCGTGTTTGTTAGCA
GTAGGGTTAACAGAAGCGTGTTTGTTAGCAGAAGGAAAGCGAGTGACGATATACACTGATTCTGCATATGCCCATAACGTATGTCATTTGTTTGGATCAGTGTGGAAAGGGCGAGGATTT
TTTGGATCAGTGTGGAAAGGGCGAGGATTTAAGAAAACGGATGGTTCTCCGATACAGCATCATGCGCAAATAATGAAACTGTTGCATGCCATGATGAAACCTAAAGAAATAGCAATAGCT
ATGATGAAACCTAAAGAAATAGCAATAGCTAAGTGTGCAGCTCATAAAACAGATGTGTCAAAAGTTACAAAAGGGAATAAAGCTGCTGATGAAGCTGCAAAAGCAATTACAGGAGCTGAC
GAAGCTGCAAAAGCAATTACAGGAGCTGACAAATTGGGTAAGGTTTTTCTGGTCACTCATGGAGTAGACCTAGAAGACAACATTACGCTTAAAGATGTGATTTTGATGCAGGAAGCTGCA
AAAGATGTGATTTTGATGCAGGAAGCTGCACCAACAATTGATAAACAGTTATGGCTAGACCGAGGTGCAGTCAAAGATTCTACTGGTCTTTGGAGAAATCATGAAGGGTTGATAGTAGCA
TGGAGAAATCATGAAGGGTTGATAGTAGCACCGCTAGACCTGCTGGGTCTGATGATCCAGGAGGCACATGGTTTAGCGCATGTTGCAAGGGGGGAAGTTAGGAGAAAGATCACAAAGGAA
GGGGAAGTTAGGAGAAAGATCACAAAGGAATATGGTTTTTGGGCACCATATTTGCTAGAACAGATTGATCACATCATAGGCAGATGTACAATTTGTTTGAAAAATAATGTTCGCCGAGGT

Figure S1

ATTTGTTTGAAAAATAATGTTCGCCGAGGTGTGACTGTTCTTCCAGGTTACATTCCAACCCCAAGAGGTCCTATGCGTGAGCTGGTTATTGACTATGTCGATATGATCAAACCAGTTGAA
GACTATGTCGATATGATCAAACCAGTTGAAGGTAAAAGATACATGCTAGTGGTTGTGGATAGATTTTCACGATGGCCGGAGGCCTGTCCAACCAAGCGGAAAGATGCTCAGTCAGTTGCC
ACCAAGCGGAAAGATGCTCAGTCAGTTGCCAAGTTTTTGTGTAGAGAGGTGATAAGCAGGTGGGGACTTCCTGATCGAATATCCTCAGATAATGGGAAAGAGTTCGTGGATAAGACAGTG
AATGGGAAAGAGTTCGTGGATAAGACAGTGAAATTGATTTTTCAAAAATTGGGAATTAAACAACGTCTTGGAGCAGTTTATCATCCACAAAGCCAAGGGATTTGCGAAAAAATGAATGGT
AGCCAAGGGATTTGCGAAAAAATGAATGGTGTTTTGAAAAATCGTATTGTCAAGATTTGCCAACATACAGGGCTGAACTGGATAGCCGCATTACCTTTAGCCTTGATGGTGTGTCGCTCA
TTACCTTTAGCCTTGATGGTGTGTCGCTCAAGTGAGTTGCGTGATTTACGTATGACACCCCATGAATTGGTCACAGGAAGACGGATGCCTACGCCTTGTCTGCGAACAAGCGGAAAAGGT
ACGCCTTGTCTGCGAACAAGCGGAAAAGGTCCAAGTTTGGCTTTTTTGGAAGATGAAATGAGAGCATATGTTACATATATGGCTAATTTCCATAAAAGAATATCCACATATGTTTCTGAC
CATAAAAGAATATCCACATATGTTTCTGACAGGCAAAGAAAAGAAGAGGTGCAGGAGAAGCTCGATGAGCAAAAAAGGAGTACAGTGCAACCTGGGGACAAGGTGTTCGTGAAGGTATTT
CCTGGGGACAAGGTGTTCGTGAAGGTATTTAGAAGGAAATGGTATAACGAACGCCGTGAAGGACCATTTGAGGTTGTTCGCAGTACGGGAACAGCCGTTCAGGTTAAAGGGTCTCCAACG
ACAGCCGTTCAGGTTAAAGGGTCTCCAACGTGGTATCATTTATCGAATTGTGTTAAAGCTCCCAGGGAAGAAACACCACGGCTAGGGAATCAAGATGTTGAGGGCTCAAAAGAGCCAGAA
CAAGATGTTGAGGGCTCAAAAGAGCCAGAAGAAAGTAGGGAAGAACAGCCAGCAGAAAGAGAGATTGTCCATAATGTGGACAATGCTGATGATCATGTGTATACTTCTGAGGATGCCAGA
GATCATGTGTATACTTCTGAGGATGCCAGACATGACACAGCAATTGATGGACAGGAAAGGAGATTTGGGGATATTGAGTTTCAATATGTCCCAGAAACAGCAGGGACTATCCCAGTGGAA
CCAGAAACAGCAGGGACTATCCCAGTGGAAGGTGAAACCACAGAGGTTGCCGAGGGCTGTAACTCKGTTACAGGAGAAGCCGGTGATTCAGTTAGACGAAATAGCCCAAGYCCAGTCAGG
GTTAGACGAAATAGCCCAAGYCCAGTCAGGAAAAAAGTCAAACCAAGACGCTACGAAGATTAGAGTCGAAGTYTCTCTAGGAAAGCCAGCTCAGCTTCCATGCCAGTGTATGAGAGYGAA
TCAGCTTCCATGCCAGTGTATGAGAGYGAATAGRGGTGAAGGAYAATTTAGAGTTCGGTGGGAAYACAGRCATGGTGGAGTTTTAGACCTGTTAGGAACAATGACATCAAGAAAGTATGC
GTTAGGAACAATGACATCAAGAAAGTATGCGTTATTAAATGATAAGAGAAGATTGAAAATTGAAGGGGATTGTAGTCTTTATGTGTATAATTCCCAACGGGAGGATCAAGGTGATTATAA
TTCCCAACGGGAGGATCAAGGTGATTATAAGTGTATTTTTTATGATCCACAGTTTGAAAGCGATGGAATAGAGTCGGGACTCAGAGTTAATGTGGTGACGCTGGTAGTAAAAGATGGGAA
TGTGGTGACGCTGGTAGTAAAAGATGGGAACAAAAGCATGGGGGTCCAAGCTGTTGAAGAAACAAAAGAATCAACAACACTGATATCAACACTTCAGCCTAACATTAGAACAATGAGTGG
ACTTCAGCCTAACATTAGAACAATGAGTGGTAATTCAACTCTTTTAGCATTGGTAGTTGCTAAAGGTATAAATGTATCAAAGTTGACTACAGTGGCGCCTCGGATGACTTCGC
AGTGGCGCCTCGGATGACTTCGC

Figure S2

RNaseH

```
fish          DGSCYRVGDK-LSAGYAVVKAQGT-GFVVEKAEVIPQPASAQLAELVGLTEACLLAEGKR
ZFERV-2       ...S..DHLG.VHT.F..I.KEKD.N..PVISQQCV..C.......KA..A..Q..K.QT
ASSBSV        ..C...GK.G.NI.A....QQDSNGNHSTLESG............II...R.LT.....T
ZFERV         ..C.FKTDSGK.V.S..I.EQTDD..YTIREQQ.LQDRP...R...LA.VR.LHM.KD.T

fish          VTIYTDSAYAHNVCHLFGSVWKGRGFKKTDGSPIQHHAQIMKLLHAMMKPKEIAIAKCAA
ZFERV-2       AN.........G......A...Q.............AE..GQ.IS...Q..RL.VI..Q.
ASSBSV        .N.........GAV.ID.PQ.LR.N.TT.GNL..K.KT.MEV.IS.VAL..KV..M..KG
ZFERV         .N.HS.....VGAATSELTG.ARV..VTSS.K..K.AQEASD..ESI.L.Q.V..I....

fish          HKTDVSKVTKGNKAADEAAK
ZFERV-2       ..KGNYL.I...N...SE..
ASSBSV        .QVLN.RIS...D...Q...
ZFERV         .TKGKDP.SL..E...A...
```

Integrase

```
fish          HGLAHVARGEVRRKITKEYGFWAPYLLEQIDHIIGRCTICLKNNVRRGVTVLPGYIPTPR
ZFERV-2       ..FD.CSK...I...KQQ-.Y.S...NAM.SEFLSK.E..A.H.I.K.TATPISH..V.E
ZFERV         .S...SSEKDMTK--RVS-QW.H.FMPHM.SGV.AS.QT.AEF..KPTSKPTA.HF..D.
ASSBSV        ...T.EGKLKTLQ--RVS.TW.H..MK.MT.LFCDN....GNY.PKKPYQTPM.HY.V.N

fish          GPMRELVIDYVDM-IKPVEG-KRYMLVVVDRFSRWPEACPTKRKDAQSVAKFLCREVISR
ZFERV-2       ..FKH..M........R.Q.........I......V..V.SADQG.GT.I...T..I.P.
ZFERV         ..GCTV.M.FT....TR.N.....L..L..Q.TG....F.CA.E..V..V.C.INQY.P.
ASSBSV        ACFQDIS...T..GADQ.R.G...L..M.......V..I..AKE..K..I.W.QT.L.P.

fish          WGLPDRISSDNGKEFVDKTVKLIFQKLGIKQRLGAVYHPQSQGICEKMNGVLKNRIVKIC
ZFERV-2       F.I.SE......SA.IQ....QVL.Q.R......CI.......MV.RV..TI.AKLN...
ZFERV         H.F.RI.R....TH.KNEHLADVEKL....L.Y..........KV.RL.LT...KLA...
ASSBSV        Y.V.RQ.R....SH.SNQHLRQVEERF..VHKF.S..K.....LV.RC.QT..AK.A.V.

fish          QHTGLNWIAALPLALMVCRSSELRDLRMTPHELVTGRRMPTPCLRTSGKGPSLAFLEDEM
ZFERV-2       AS.N...VD.......SY.MQTNKHTSN.-A.ML..CP..V.FC.GPY.E.P.EQ.QM.L
ZFERV         HKSK...VD...I...SV.C.IN.TTGF..F..A...QF.G--PVAPLHAGDTDSPQM--
ASSBSV        AG.K.T.VE.......AM...PGAGTHLS...IM...V..G.-P.EG.HM.A.DVHQIG.

fish          RAYVTYMANFHKRISTYVSDRQRKEEVQEKLDEQKRSTVQPGDKVFVKVFRRKWYNERRE
ZFERV-2       .S.MKNLTAI..--AI..QETRKG--.R.EVETP--GP.V...Q.YL.......NEA...
ZFERV         --.HDKVCAVINMF.PQK.WPTES----.AS----.PAENTTLW.RL.QHK...SSP.WS
ASSBSV        TD..RKLTELSAAL.IQIQRVAEG----.LT.D.DQPR.KV..W.RI..HK...ADP.WT

fish          GPFEVVRSTGTAVQVKG
ZFERV-2       ..YR.I.A.P.....E.
ZFERV         E.LR.TAR.SHC..LA.
ASSBSV        ..Y..KEV.SHS.....
```

Figure S3

gag

```
lion        MGQTQTTPLSIMIDHFKDVRGRANNLSVEVRKGRWQFFCSSEWPTFNVGWPPEGTFDLPT
AY364318    ....V.....LTLN.WSE.QA..R.QG.....KK.ITL.EA..VMM.....R....TIDN
AY364319    ....V.....LTLN.WSE.QA..R.QG.....KK.ITL.EA..VMM.....R....TIDN
FeLV        ....I.....LTL..WSE..A..H.QG.....KK.ITL.EA..VMM.....R....S.DN

lion        IHRVRSIISQP-KTGHLDQLPYIITWQDLVEDPPSWLKPFLAPLPPEPKPILALQGTKKK
AY364318    .SQ.EER.FA.GPY..P..I...T..RS.AT...P.VR...P.PKHPRTDPPEPLSPQPS
AY364319    .SQ.EER.FA.GPY..P..I...T..RS.AT...P.VR...P.PKHPRTDPPEPLSPQPS
FeLV        .SQ.EKK.FA.GPY..P..V...T..RS.AT.....VR...P.PK.P-T.LPQPLSPQPS

lion        KSLIQPSAPLYPVLQGGTEEELIFPPSYNPSRMLEEHHPPPPGEADAVPRAGGGNAPVGS
AY364318    APP.SSLY.VL.KPDPPKAPV.PPN..SPLIDL.T.EP..Y..GHGPT.PS.P-RT.TA.
AY364319    APP.SSLY.VL.KPDPPKAPV.PPN..SPLIDL.T.EP..Y..GHGPT.PS.P-RT.TA.
FeLV        AP.TSSLY.VL.KSDPPKPPV.PPD..SPLIDL.T.EP..Y..GHGP-.PS.P-RT.TA.

lion        PPFTRQRAQREQSASAADSTILPLRATGPPDAEGNQPHHYWPFATSDLYNWKAQNPKFSE
AY364318    .IAS.L.ER..N--P.EK.QA....E-..-----.NRPQ....SA.......LH..P..Q
AY364319    .IAS.L.ER..N--P.EK.QA....E-..-----.NRPQ....SA.......LH..P..Q
FeLV        .IAS.L.ER..N--P.EE.QA....E-..-----.NRPQ....SA.......SH..P..Q

lion        KPAGLIDLLDSVLFTHQPTWDDCQQLLQVLFTTEERERIVNEARKLVPGTDGNPTTNQAQ
AY364318    D.VA.TN.IE.I.V.............A.L.A...Q.VLL....Q...E..R..QLPNV
AY364319    D.VA.TN.IE.I.V.............A.L.A...Q.VLL....Q...E..R..QLPNV
FeLV        D.VA.TN.IE.I.V.............A.L.G...Q.VLL....Q...E..R..QLPNV

lion        IDASFPLTRPQWDFNTAEGKERLRVYRQTLMGGLRMAARKPTNLAKVGNVQQGKDESPAA
AY364318    V.EA......N...A.PA.R.H..L...L.LA...G...R.....Q.KQ.V...E.T..S
AY364319    V.EA......N...A.PA.R.H..L...L.LA...G...R.....Q.KQ.V...E.T..S
FeLV        ..ET......N...A.PA.R.H..L...L.LA...G...R.....Q.KQ.V...E.T...

lion        FLERIMEAFRTYTPMDPEAPESKAAVIMAFVNQSAIDIRRKLQKIDRLGEKSLQDLLVVA
AY364318    ....LK..Y.M...Y...D.GQA.S..LS.IY..SP...N...RLEG.QGFT.S...KE.
AY364319    ....LK..Y.M...Y...D.GQA.S..LS.IY..SP...N...RLEG.QGFT.S...KE.
FeLV        ....LK..Y.M...Y...D.GQA.S..LS.IY..SP...N...RLEG.QGFT.S...KE.

lion        EKVYNNRELPEDKQARAMAAASS---KQTRDLARILLATTADSPEERDRRLWQLADDARK
AY364318    ..I..K..T..EREE.LWQRQEERDK.RHKEMTKV.ATVV.QN-RDK..EESK.G.QR--
AY364319    ..I..K..T..EREE.LWQRQEERDK.RHKEMTKV.ATVV.QN-RDK..EESK.G.QR--
FeLV        ..I..K..T..EREE.LWQRQEERDK.RHKEMTKV.ATVV.QN-RDK..EESK.G.QR--

lion        GKRTTKGGKQRLQKDQCAYCKEIGHWARDCLKRAGGKGSKTDRV
AY364318    --------.IP.G.........K...V...P..PRK.PADSTLL
AY364319    --------.IP.G.........K...V...P..PRK.PADSTLL
FeLV        --------.IP.G.........K...V...P..PRK.PANSTLL
```

pol

```
lion        ELSD-GSRGSDPLPEPRVTLKVEGTPVDFLVDTGAQHSVLRTPQGKLASKKSWVQGATGM
AY364318    N.E..E.Q.Q..P....I....G.Q..T...........TR.D.P.SDRSAL......S
AY364319    N.E..E.Q.Q..P....I....G.Q..T...........TR.D.P.SDRSAL......S
FeLV        N.G.QE.Q.Q..P....I...IG.Q..T...........TR.D.P.SDRTAL......S

lion        SQYSWTTRRTVDLGTGRVSHSFMVIPECPYPLLGRDLLTKIGAQITFRQGGPQVTDGKGH
AY364318    KN.R...D.R.Q.A..K.T...LYV..............LK...H.TGE.AN.VGPM.L
AY364319    KN.R...D.R.Q.A..K.T...LYV..............LK...H.TGE.AN.VGPM.L
FeLV        KN.R...D.R.Q.A..K.T...LYV..............LK...H.TGE.AN.VGP..L

lion        PIQVLTMKLEDEYLLHQEALPREDNIDRWLQEFPSVWAETGGGMGLAAHRTPVLVELKPG
AY364318    .L....LQ..E..R.FEPESELKQGM.S..KN..QA......-I.M.HCQA.I.IQ..AT
AY364319    .L....LQ..E..R.FEPESELKQGM.S..KN..QA......-I.M.HCQA.I.IQ..AT
FeLV        .L....LQ..E..R.FEPESTQKQEM.I..KN..QA......-..T.HCQA...IQ..AT
```

Figure S3

```
lion        ESPVRIKQYPMSQEARKGIQPHIRRLRSLGVLVPCQSAWNTPLLPVKKPHTNDYRPVQDL
AY364318    AT.IS.R....PH..YQ..K.....MLDQ.I.K....P...........G.G........
AY364319    AT.IS.R....PH..YQ..K.....MLDQ.I.K....P...........G.G........
FeLV        AT.IS.R....PH..YQ..K.....MLDQ.I.K....P...........G.E........

lion        REVNKRVADIHPTVPNPYTLLSSLAPSRVWYTVLDLKDAFFSLPLAPQSQPLFAFEWHDP
AY364318    .......E..........N...T.P..HP............C.R.H.E..L......K..
AY364319    .......E..........N...T.P..HP............C.R.H.E..L......K..
FeLV        .......E..........N...T.P..HP............C.R.HSE..L......R..

lion        EEGYSGQLTWTRLPQGFKNSPTIFDEALHEDLGEYRREHPGLTLLQYVDDILIAADTAKD
AY364318    .I.L..................L......S..ADF.VRY.A.V.......L.L..A.KTE
AY364319    .I.L..................L......S..ADF.VRY.A.V.......L.L..A.KTE
FeLV        .I.L..................L......S..ADF.VRY.A.V.......L.L..A.RTE

lion        CERGTQDLLATLGALGYRASAKKAQICRERVSYLGYILEGGQRRLSDARKETVLKIPTPT
AY364318    .LE..KA..E...NK............LQK.T....S..D...W.TK....AI.S..V.K
AY364319    .LE..KA..E...NK............LQK.T....S..D...W.TK....AI.S..V.K
FeLV        .LE..KA..E...NK............LQE.T....S.KD...W.TK....AI.S..V.K

lion        SRREVREFLGSAGYCRLWVPGFAEIARPLYEATKEGKTFKWTEKEEIAFNQLKKALLSAP
AY364318    NP.Q......T.......I.....L.A...PL.RP.TL.Q.GTEQQL..ENI......S.
AY364319    NP.Q......T.......I.....L.A...PL.RP.TL.Q.GTEQQL..ENI......S.
FeLV        NS.Q......T.......I.....L.A...PL.RP.TL.Q.GTEQQL..EDI......S.

lion        ALGLPDIMKPFHLFVDEHKGIAKGVLTQALGPWNRPVAYLSKKLDPVAAGWPPCLRIIAA
AY364318    .......T...E..I..SS.F.....V.K....K...........T..S.......MV..
AY364319    .......T...E..I..SS.F.....V.K....K...........T..S.......MV..
FeLV        .......T...E..I..NS.F.....V.K....K...........T..S.......MV..

lion        TALLVKDADKLTLGQEIWITTPHAIEGVLKQPPDRWMSNTRVTHYQSLLLNPPRVRFHPS
AY364318    I.I.....G......PLTVL.S.PV.ALVR...NK.L..A.M....AM..DAE..H.G.T
AY364319    I.I.....G......QLTVL.S.PV.ALVR...NK.L..A.M....AM..DAE..H.G.T
FeLV        I.I.....G......PLT.L.S.PV.ALVR...NK.L..A.M....AM..DAE..H.G.T

lion        AALNPATLLPDPDLGAPLHDCAGILEQVHGFRMDLTDQPLPDAEATWFTDGSSFVRDGHR
AY364318    VS........L.NE.N-H...LQ..AET..T.P..........DL..Y......I.N.E.
AY364319    VS........L.NE.N-H...LQ..AET..T.P..........DL..Y......I.N.E.
FeLV        VS........L.SG.N-H...LQ..AET..T.P..........DL..Y......I.N.E.

lion        YAGAAVVTEMDTVWAEALPSGTSAQRAELIALTKALMLGAGKRLNIYTDSRYAFATAHIH
AY364318    K.....T..SEVI..AS..P.............Q..KMAK..K.TV............V.
AY364319    K.....T..SEVI..AS..P.............Q..KMAK..K.TV............V.
FeLV        E.....T..SEVI..AP..P.............Q..KMAE..K.TV..........T.V.

lion        GAIYQERGLLTAEGRTIKNKQEILNLLTALWLPAKLAIIHCQGHQKADNPVARGNRKADQ
AY364318    .E..RR.....S..KE....N...A..E..F..KR.S....P....G.S.Q.K...L..D
AY364319    .E..RR.....S..KE....N...A..E..F..KR.S....P....G.S.Q.K...L..D
FeLV        .E..RR.....S..KE....N...A..E..F..KR.S....P....G.S.Q.K...L..D

lion        AAKAVALTSVPTMTIQLPDPGDPVLPDQPKYSQEELQRIKKLPMAQEIKGWWYTPNKELV
AY364318    T..KA.TETQSSL..LPTELIEGPKRPPWE.DDSD.DLVQ..EAHY.P.RGTWEYQGKTI
AY364319    T..KA.TETQSSL..LPTELIEGPKRPPWE.DDSD.DLVQ..EAHY.P.RGTWEYQGKTI
FeLV        T..KA.TETHSSL.VLPTELIEGPKRPPWE.DDSD.DLVQ..EAHY.P.RGTWEYRGKTI

lion        LPDRLGVSILEHMHRSTHMGARKLKDLIR--HAGIKIHQQDTKIEQVVSACKTCQLTNAK
AY364318    M.EKYAKELIS.L.KL..LS...M.T.LEREET.FYLPNR.LHLR..TES.RA.AQI..G
AY364319    M.EKYAKELIS.L.KL..LS...M.T.LEREET.FYLPNR.LHLR..TES.RA.AQI..G
FeLV        M.EKYAKELIS.L.KL..LS...M.T.LEREET.FYLPNR.LHLR..TES.RA.AQI..G

lion        ATSNKKGTRLRGTRPGAQWEVDFTEVKPGKYGYKYLLVFTDTFSGWVEAYPTKHETAQTV
AY364318    KIKFGPDV.A..H...IH.......I...M.........I......A....A.....KV.
AY364319    KIKFGPDV.A..H...IH.......I...M.........I......A....A.....KV.
FeLV        KIKFGPDV.A..R...TH.......I...M.........I......A....A.....KV.
```

Figure S3

```
lion          AKKLLEDILPRYGFPAMVGSDNGPAFISQVTQAVAKAVGANWKLHCAYRPQSSGQVERMN
AY364318      ......E.F....I.QVL............S.S..TLL.I....................
AY364319      ......E.F....I.QVL............S.S..TLL.I....................
FeLV          ......E.F....I.QVL............S.S..TLL.I....................

lion          RTLKETLTKLTMETGG-DWVTLLPYALYRVRNTPYTLGFTPYEIMFGRPPPVIPSLRAEL
AY364318      .SI........L...SK...L...LV........GPH.L..F..LY.A...MAHFFD.DI
AY364319      .SI........L...SK...L...LV........GPH.L..F..LY.A...MAHFFD.DI
FeLV          .SI........L...SK...L...LV........GPH.L..F..LY.A...MAHFFDTDI

lion          LAEFKDQELFLSLRGLQRAHEDIWPRLRAIYEAGPTP--TPHQYKPGDWVYVKRHHRETL
AY364318      SGFATSPTMQAH..A..LVQ.E.QRP.A.A.REKLETPVV..PF....S.W.R..QTKN.
AY364319      SGFATSPTMQAH..A..LVQ.E.QRP.A.A.REKLETPVV..PF....S.W.R..QTKN.
FeLV          SSFATSPTMQAH..A..LVQ.E.QRP.A.A.REKLETPVV..PF....S.W.R..QTKN.

lion          EPRWKGPYIVVLTTPTALKVDGIATWVHHTHVRPADPSSIRK
AY364318      .......H..L...........V.A.I.AS..KA.G.TTNQD
AY364319      .......H..L...........V.A.I.AS..KA.G.TTNQD
FeLV          .......H..L...........V.A.I.AS..KA.G.TTNQD
```

Figure S4

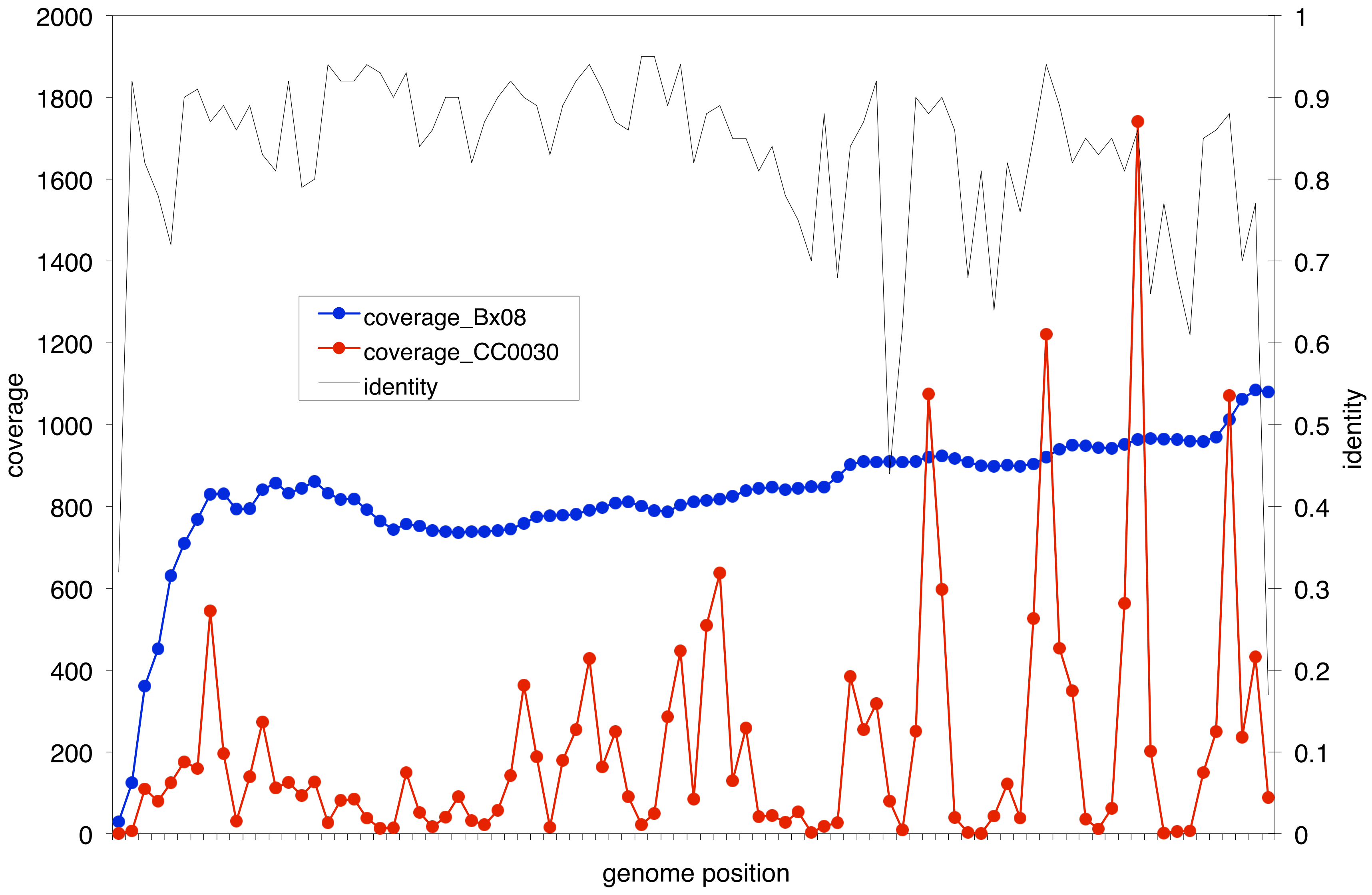

2000
1800
1600
1400
1200
1000
800
600
400
200
0
coverage
1
0.9
0.8
0.7
0.6
0.5
0.4
0.3
0.2
0.1
0
identity
coverage_Bx08
coverage_CC0030
identity
genome position

Figure S5

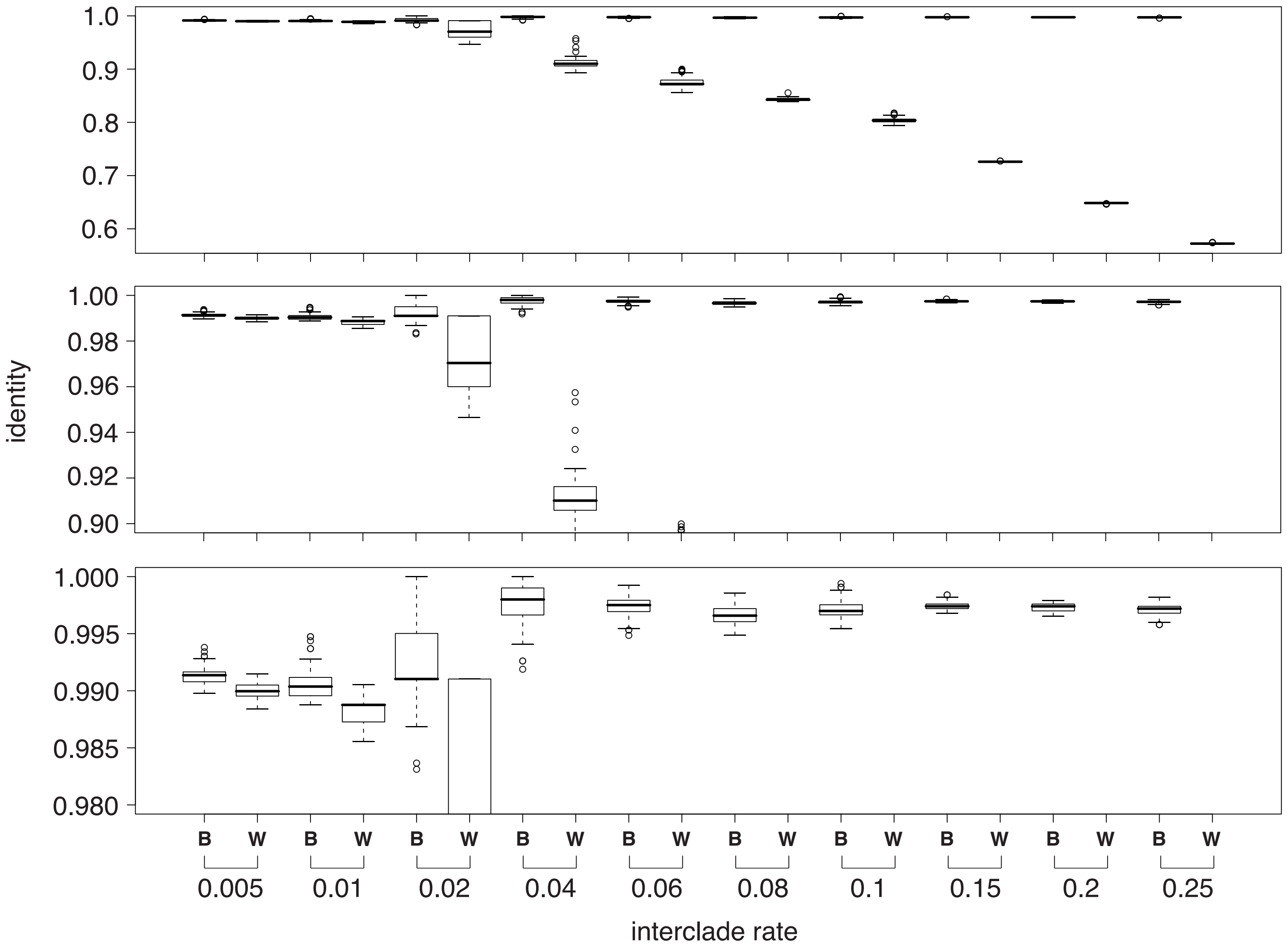

1.0
0.9
0.8
0.7
0.6
1.00
0.98
0.96
0.94
0.92
0.90
1.000
0.995
0.990
0.985
0.980
identity
B W B W B W B W B W B W B W B W B W B W
0.005 0.01 0.02 0.04 0.06 0.08 0.1 0.15 0.2 0.25
interclade rate

Figure S6

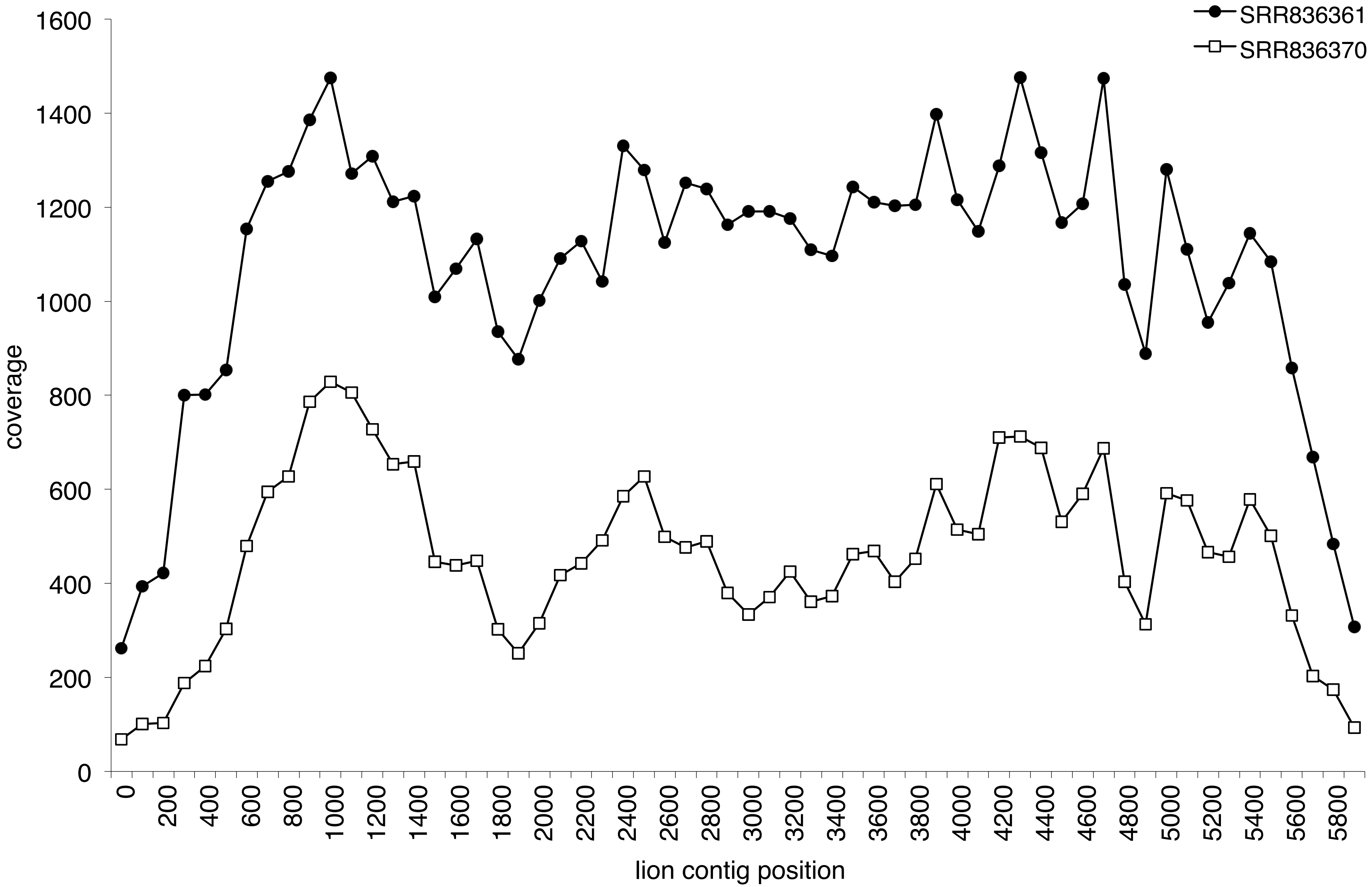

SRR836361
SRR836370
coverage
1600
1400
1200
1000
800
600
400
200
0
0
200
400
600
800
1000
1200
1400
1600
1800
2000
2200
2400
2600
2800
3000
3200
3400
3600
3800
4000
4200
4400
4600
4800
5000
5200
5400
5600
5800
lion contig position

Table S1. Read coverage

	contig/consensus size	total positions covered	average coverage	position with coverage ≥5 reads	position with coverage ≥5 reads, percentage
perch1	2903	2846	13.7	2748	94.7
perch2	2903	2849	10.3	2323	80.0
perch3	2903	2868	19.1	2618	90.2
perch4	2903	2893	24.5	2761	95.1
lion1	5976	5971	37.8	5824	97.5
lion2	5976	5976	17.4	5800	97.1
lion3	5976	5975	72.1	5608	93.8
lion4	5976	5972	35.5	5778	96.7
HERVK	9472	9416	27.4	6729	71.0
HERVH	5826	5428	46.3	4379	75.2
HERVW	2781	2354	7.5	698	25.1
Bx08	8735	8735	1053.9	8607	98.5
CC0030	8884	8514	211.1	7882	88.7
dual.Bx08	8735	8734	219.6	8675	99.3
dual.CC0300	8884	8514	212.7	7881	88.7
